# Case of the Cure of Pertussis

**Published:** 1802-06

**Authors:** H. Davies

**Affiliations:** Piccadilly


### *Case of the Cure of Pertuffis communicated by Mr.* H. Davies, *of Piccadilly.*

THE Hooping Cough, of late, has been very prevalent both in the metropolis and adjacent parts. In many inftances it has proved fatal, in all diftreffing; every improvement therefore in the plan of treating this difeafe, by fhortening the period and alleviating its fymptoms, muft prove a valuable acquifition to the healing art, afford a fource of fatisfaction to the practitioner, as well as comfort and happinefs to the patient,

I was pleafed on obferving in your laft Journal, an account of the beneficial effects attending the external ufe of Opium in this difeafe, and fhall be happy if, upon future trials, its utility be confirmed and eftablifhed.

I beg leave to mention an inftance in my own practice, where an exhibition of the aqueous folution of Afafœtida, joined with the decoct. cinchon. proved highly beneficial in this difeafe. A few weeks fince, my attendance was requefted in a family in Oxford-ftreet, where five of the children were attacked with the hooping cough; the youngeft of whom was but *five weeks* old, the ages of the reft were from three to nine. I began the treatment by adminiftering an antimonial emetic, which was repeated every third or fourth day; and on the intermediate days, ordered the folution with an equal part of the decoction of yellow bark to be given three times a day, in dofes fuited to their refpective ages. But, being apprehenfive that the medicine was fomewhat too noxious to be retained on the ftomach of the infant, on account of its tender age, I fubftituted fmall dofes of the extr. cicutæ in a pleafant mixture, with the addition of a few drops of the vin. antim. tartar. and blifters applied to the fternum, with confiderable advantage; infomuch, that the paroxyfms, which threatened immediate fuffocation, were in the courfe of a few days furprifingly abated, and in two or three weeks the infant entirely recovered.

The other children perfifted regularly in the ufe of the folution and bark; which produced the happieft effects in diminifhing ing the ſpaſmodic tendency of the diſeaſe, and in about the ſame ſpace of time they became convaleſcents; experiencing only a ſlight trivial cough now and then, which perhaps might be deemed (according to Dr. Cullen) merely the effect of habit in the conſtitution, as a conſequence of the former malady.

I have been long prepoſſeſſed in favor of the antiſpaſmodic effects of aſafœtida in this diſorder; but, from its noxious taſte, and the fixed averſion in children to medicines in general, eſpecially thoſe of the diſagreeable kind, I never had an opportunity of ſeeing it fairly and regularly adminiſtered, except in the inſtance now mentioned; and I am very happy in not finding my expectations diſappointed. Should a future trial of this medicine, in the hands of judicious practitioners, be attended with equal ſucceſs, it will afford further gratification; as I know no claſs of patients that lays a juſter claim to our tendereſt regard and minuteſt attention than the infantile race, whoſe diſeaſes are as numerous as they are oppreſſive to their delicate ſyſtems.

*Piccadilly, April* 9, 1802.

